# The impact of monthly air pollution exposure and its interaction with individual factors: Insight from a large cohort study of comprehensive hospitalizations in Guangzhou area

**DOI:** 10.3389/fpubh.2023.1137196

**Published:** 2023-03-21

**Authors:** Xu Ju, Wumitijiang Yimaer, Zhicheng Du, Xinran Wang, Huanle Cai, Shirui Chen, Yuqin Zhang, Gonghua Wu, Wenjing Wu, Xiao Lin, Ying Wang, Jie Jiang, Weihua Hu, Wangjian Zhang, Yuantao Hao

**Affiliations:** ^1^Department of Medical Statistics, School of Public Health & Center for Health Information Research & Sun Yat-sen Global Health Institute, Sun Yat-sen University, Guangzhou, China; ^2^Peking University Center for Public Health and Epidemic Preparedness and Response, Peking, China

**Keywords:** hospitalizations, air pollution, effect modification, time-dependent Cox proportional model, particulate matter

## Abstract

**Background:**

Although the association between short-term air pollution exposure and certain hospitalizations has been well documented, evidence on the effect of longer-term (e. g., monthly) air pollution on a comprehensive set of outcomes is still limited.

**Method:**

A total of 68,416 people in South China were enrolled and followed up during 2019–2020. Monthly air pollution level was estimated using a validated ordinary Kriging method and assigned to individuals. Time-dependent Cox models were developed to estimate the relationship between monthly PM_10_ and O_3_ exposures and the all-cause and cause-specific hospitalizations after adjusting for confounders. The interaction between air pollution and individual factors was also investigated.

**Results:**

Overall, each 10 μg/m^3^ increase in PM_10_ concentration was associated with a 3.1% (95%*CI*: 1.3%−4.9%) increment in the risk of all-cause hospitalization. The estimate was even greater following O_3_ exposure (6.8%, 5.5%−8.2%). Furthermore, each 10 μg/m^3^ increase in PM_10_ was associated with a 2.3%-9.1% elevation in all the cause-specific hospitalizations except for those related to respiratory and digestive diseases. The same increment in O_3_ was relevant to a 4.7%−22.8% elevation in the risk except for respiratory diseases. Additionally, the older individuals tended to be more vulnerable to PM_10_ exposure (*P*_interaction_: 0.002), while the alcohol abused and those with an abnormal BMI were more vulnerable to the impact of O_3_ (*P*_interaction_: 0.052 and 0.011). However, the heavy smokers were less vulnerable to O_3_ exposure (*P*_interaction_: 0.032).

**Conclusion:**

We provide comprehensive evidence on the hospitalization hazard of monthly PM_10_ and O_3_ exposure and their interaction with individual factors.

## 1. Introduction

The association between air pollution and human health has been well documented. For example, a meta-analysis on top of 21 studies worldwide suggested a 1.0% and 0.4% increase in hospitalization or emergency room visit for pneumonia for every 10 μg/m^3^ increment in the short-term exposure to PM_2.5_ and PM_10_ (1). Another meta-analysis also reported that each 10 μg/m^3^ increase in the 6-day cumulative PM_2.5_ concentration was associated with a 2.5% rise in chronic obstructive pulmonary disease hospitalization (2). Similar adverse health impact of other pollutants such as NO_2_, O_3_, and NO were also reported previously (3).

As suggested by previous studies, people of certain characteristics tended to be more vulnerable to the health impact of air pollution. For example, Michelle et al. pooled the results of 108 studies and concluded that the older individuals suffered from a greater mortality risk than the younger ones following the same exposure to particulate matters, with a 0.64% vs. 0.34% increase in mortality per 10 μg/m^3^ increment in PM_10_ concentration (4). They also observed that women had a slightly higher mortality risk of 0.55% compared with 0.50% among men (4). Compared with basic demographic variables such as age and sex, lifestyle information (e.g., smoking, drinking and physical activities), existing health status (e.g., overweight) and preexisting health conditions may be of a greater public health significance as they usually are more feasible to be modified to improve human wellbeing. The modification effect of these individual factors on the health impact of air pollution was biologically plausible (5–12). For example, smoking, alcohol consumption and air pollution were considered to increase the oxidative stress and dysfunction within cell mitochondria (5, 6). It is likely that smoking and drinking may strengthen the adverse health impact of air pollution in a synergistic way.

Although a lot of efforts have been taken to investigate the potential individual modifiers underlying the health impact of air pollution, existing evidence was challenged by several issues. First, most of existing works focused on the acute health effect of air pollution which usually occurred within one week following exposure, however, the evidence for exposures over a longer-term may be of a greater significance in policy making. Moreover, current studies only involved limited air pollutants or outcomes, systematic evidence for multiple pollutants and a comprehensive set of health outcomes is still limited. Additionally, the modifiers on the health effect of air pollution in existing studies generally were basic demographics (e.g., gender, age) or common lifestyle variables (e.g., smoking, drinking), evidence on a more comprehensive set of individual variables would be needed.

To address these research gaps, we investigated the effect of longer-term (i.e., monthly) exposure to main air pollutants on all-cause and cause-specific hospitalizations. Stratified effects and interactions were further investigated to identify potential individual effect modifiers. Our work provides a comprehensive insight into the health effect of air pollution and its modification by unhealthy lifestyles, demographics and preexisting health conditions, etc.

## 2. Methods

### 2.1. Study design and participants

We did a retrospective cohort study in Guangzhou area from 1/1/2019 through 12/31/2020 based on the primary medical and health service facilities. A multi-stage sampling was used to recruit participants from Community Healthcare Center-based population. In the first stage, 39 Community Healthcare Centers (183 in total) were randomly selected in Guangzhou. In the second stage, the residents who registered at these centers and met our inclusion criteria were invited to participate in our study voluntarily. The participants were followed up from the day entering the cohort to the first hospital admission, death or end of the study period, whichever came first. Participants followed up for less than 28 days were excluded to ensure sufficient time for exposure. Hospitalization records were retrieved from Guangzhou Health Commission. We obtained basic demographics (i.e., gender, age, ethnicity, education, retirement status, and marital status), lifestyle variables (smoking, alcohol intake, and activity level), and preexisting health conditions (BMI and disease history) through a questionnaire survey upon enrollment.

Individuals who were suffering from malignant tumor (591, 0.77%) or followed up for <28 days (9041, 11.7%) were excluded, leaving 68,416 participants in the final analysis. All-cause hospitalization was selected as the primary outcome. In addition, according to the number of patients, we also selected the top four subtypes as secondary outcomes including the hospitalizations due to (1) circulatory system disease; (2) respiratory system diseases; (3) digestive system disease; and (4) endocrine, nutritional and metabolic diseases with ICD-10 (International Statistical Classification of Diseases and Related Health Problems, 10th Revision) codes of I00-I99, J00-J99, K00-K93 and E00-E90, respectively. This study was approved by the School of Public Health, Sun Yat-sen University. All individuals consented to be included in the study.

### 2.2. Exposure assessment

Data on the concentration of four main air pollutants (i.e., PM_10_, PM_2.5_, NO_2_, and O_3_) were retrieved from the China National Environmental Monitoring Centre (https://air.cnemc.cn:18007). The monthly concentration of air pollution in Guangzhou during the study period was generated based on data collected from 98 monitoring stations in Guangdong through the ordinary Kriging interpolation method at a 0.01° × 0.01° resolution. The 10-fold cross-validation was performed to ensure the accuracy of the estimates, with the mean absolute percentage error (MAPE), root mean square error (RMSE) and determination coefficient (*R*-square) being computed and described in Supplementary Table S1, which showed similar accuracy with existing studies (13, 14).

As the residential address for each participant was not available while a community was relatively small, we used the coordinates (i.e., longitude and latitude) of the health service center in each community as a surrogate of the residential location. The coordinates were requested through the AutoNavi Map web service API (15, 16) (https://lbs.amap.com/). The monthly air pollution concentrations at each coordinate were assigned to the participants in the corresponding community and were further incorporated as a time-varying variable in the analysis.

### 2.3. Statistical analysis

We performed time-dependent Cox proportional hazard models to estimate the effect of long-term air pollution on both all-cause and specific-cause hospitalizations. Compared with the traditional Cox models, the time-dependent Cox models are more reliable against the proportional hazard assumption. Specifically, we established four models with an increased number of covariates being adjusted. Model 1 was a crude model which only included two air pollutants (i.e., PM_10_ and O_3_) without strong correlations (Pearson's rho < |0.8|, Supplementary Table S2). Other pollutants were excluded due to the collinearity issue. In model 2, we additionally adjusted for all basic demographics including age, gender, education, marital status, and retirement status. In model 3, we additionally included three lifestyle variables and BMI, and in model 4 we additionally adjusted for preexisting health conditions (i.e., hypertension, diabetes, and hyperlipidemia).

To evaluate whether effects of PM_10_ and O_3_ were modified by individual factors and to identify the most susceptible subgroups, we performed a comprehensive set of stratified analyses. Smoking status was measured by the smoking index (BI, BI = the number of cigarettes smoked per day × the duration of smoking in years), and was classified as never, light (BI < 400) and heavy (BI ≥ 400) according to previous studies (17–19). We defined the alcohol intake into never, under limit and over limit according to the Chinese Dietary Guidelines 2016 with limits of 25 g/day and 15 g/day for men and women, respectively (20). The activity level was categorized with the same method as literature, and comprised of 3 types (i.e., light, moderate, and heavy) (21). Participants were also grouped according to BMI (i.e., underweight: <18.5 kg/m^2^; normal: 18.5–23.9 kg/m^2^; overweight: 24–27.9 kg/m^2^; obese: >28 kg/m^2^). *P* for interaction was computed by including an interaction term of a certain pair of air pollution and individual factor. We also conducted a sensitive analysis by using PM_2.5_ instead of PM_10_. All hazard ratios (*HR*) were reported for each 10 μg/m^3^ increase in concentrations of air pollution. We used R software version 4.1.3 for our analyses.

## 3. Results

### 3.1. Descriptive statistics

A total of 68,416 participants were included in our study. The characteristics of population including basic demographics, lifestyle variables, preexisting health conditions are presented in Table 1. Over the study period, 5,278 hospital admissions were reported which accounted for 7.72% of the total population. Among the whole group, 40,212 (58.80%) were older individuals, 40,682 (59.50%) were female, 67,563 (98.80%) were Han Chinese, 59,259 (86.60%) never smoked, 61,580 (90.00%) never drank and 28,636 (41.80%) were with moderate level of physical activities. Across the whole study period, the daily average concentrations of PM_10_ and O_3_ in Guangzhou were 49.28 μg/m^3^ and 99.46 μg/m^3^, respectively.

**Table 1:** Characteristics of the study population (*N* = 68,416).

**Characteristics**	**Categories**	***n***	**Rate (%)**
**Cause of hospitalization**	All cause	5,278	7.72
	Circulatory system disease	1,371	2.00
	Respiratory system diseases	384	0.56
	Digestive system disease	485	0.71
	Endocrine, nutritional and metabolic diseases	280	0.41
**Basic demographics**			
Age	≤65	28,204	41.20
	>65	40,212	58.80
Gender	Male	27,734	40.50
	Female	40,682	59.50
Ethnicity	Han	67,563	98.80
	Others	853	1.20
Education	Illiterate or primary	37,366	54.60
	Secondary	23,180	33.90
	College or higher	7,870	11.50
Retired	Yes	3,255	4.80
	No	65,161	95.20
Marital status	Single	14,377	21.00
	Married	48,998	71.60
	Widowed	3,854	5.60
	Divorced or separated	1,187	1.70
**Lifestyle information**			
Smoking status	Never	59,259	86.60
	Light	2,050	3.00
	Heavy	7,107	10.40
Alcohol intake	Never	61,580	90.00
	Under limit	5,799	8.50
	Over limit	1,037	1.50
Activity level	Light	23,663	34.60
	Moderate	28,636	41.80
	Heavy	16,117	23.60
**Preexisting health condition**			
BMI	Underweight	6,781	9.90
	Normal	38,427	56.20
	Overweight	17,985	26.30
	Obese	5,223	7.60
Hypertension	No	46,339	67.70
	Yes	22,077	32.30
Diabetes	No	60,271	88.10
	Yes	8,145	11.90
Hyperlipidemia	No	54,756	80.00
	Yes	13,660	20.00

Supplementary Table S3 shows the result of univariate analysis. The association between the risk of all-cause hospital admission and O_3_, PM_10_, age, gender, education level, marital status, retirement status, alcohol intake, activity level, BMI and disease history were statistically significant (*P* < 0.05).

### 3.2. Association between air pollution and hospital admissions

Table 2 shows the association between air pollution and all-cause hospital admissions in single-pollutant and two-pollutant models. Effect estimates were generally consistent between the two sets of models as well as across the models with sequential adjustment of covariates. According to the final two-pollutant models, each 10 μg/m^3^ increase in the concentration of O_3_ and PM_10_ was associated with a 6.8% higher hazard (*HR*, 1.068; 95% *CI*, 1.055, 1.082) and a 3.1% higher hazard of the all-cause hospital admissions (*HR*, 1.031; 95% *CI*, 1.013, 1.049), respectively. When we used PM_2.5_ instead of PM_10_, we found the changes in the results for O_3_ were subtle, and we did not observe any significant effect for PM_2.5_ (Supplementary Table S4).

**Table 2:** Association between air pollution and all-cause hospitalization risk.

	**Single O_3_ model**	**Single PM_10_ model**	**Two-pollutant model**	
			**O**_3_	**PM**_10_
	***HR*** **(95%** ***CI*****)**	***HR*** **(95%** ***CI*****)**	***HR*** **(95%** ***CI*****)**	***HR*** **(95%** ***CI*****)**
Model 1	1.081 (1.067,1.095)	1.023 (1.005,1.041)	1.081 (1.067,1.094)	1.022 (1.004,1.040)
Model 2	1.071 (1.058,1.085)	1.033 (1.015,1.051)	1.070 (1.056,1.083)	1.030 (1.012,1.048)
Model 3	1.071 (1.057,1.085)	1.033 (1.015,1.051)	1.070 (1.056,1.083)	1.030 (1.012,1.048)
Model 4	1.069 (1.056,1.083)	1.034 (1.016,1.052)	1.068 (1.055,1.082)	1.031 (1.013,1.049)

Model 1: crude two-pollutant model; Model 2: adjusted by basic demographics; Model 3: Model 2 + lifestyle information and BMI; Model 4: Model 3 + other preexisting health conditions.

When the results were stratified by the cause of hospital admissions (Table 3), we found that each 10 μg/m^3^ increase in the concentration of O_3_ was associated with a 4.7%-22.8% increase in the risk of hospital admissions due to one of the multiple outcomes except for the respiratory diseases for which no significant signals were detected. Similarly, we found that each 10 μg/m^3^ increase in the concentration of PM_10_ was related to a 2.3%−8.1% increase in the risk of hospital admissions due to reasons other than respiratory and digestive diseases. Results were also consistent between the single-pollutant and two-pollutant models.

**Table 3:** Association between air pollution and cause-specific hospitalization risk.

**Specific cause**	**Single O_3_ model**	**Single PM_10_ model**	**Two-pollutant model**	
			**O**_3_	**PM**_10_
	***HR*** **(95%** ***CI*****)**	***HR*** **(95%** ***CI*****)**	***HR*** **(95%** ***CI*****)**	***HR*** **(95%** ***CI*****)**
Circulatory system diseases	1.048 (1.022,1.074)	1.025 (0.991,1.061)	1.047 (1.022,1.073)	1.023 (0.989,1.058)
Respiratory system diseases	0.985 (0.940,1.031)	1.080 (1.015,1.149)	0.984 (0.940,1.029)	1.081 (1.016,1.149)
Digestive system diseases	1.104 (1.059,1.152)	1.069 (1.010,1.132)	1.100 (1.056,1.147)	1.064 (1.004,1.128)
Endocrine, nutritional and metabolic diseases	1.109 (1.051,1.172)	0.981 (0.909,1.059)	1.111 (1.052,1.174)	0.974 (0.901,1.053)

### 3.3. Moderating effect

**Figure 1:**
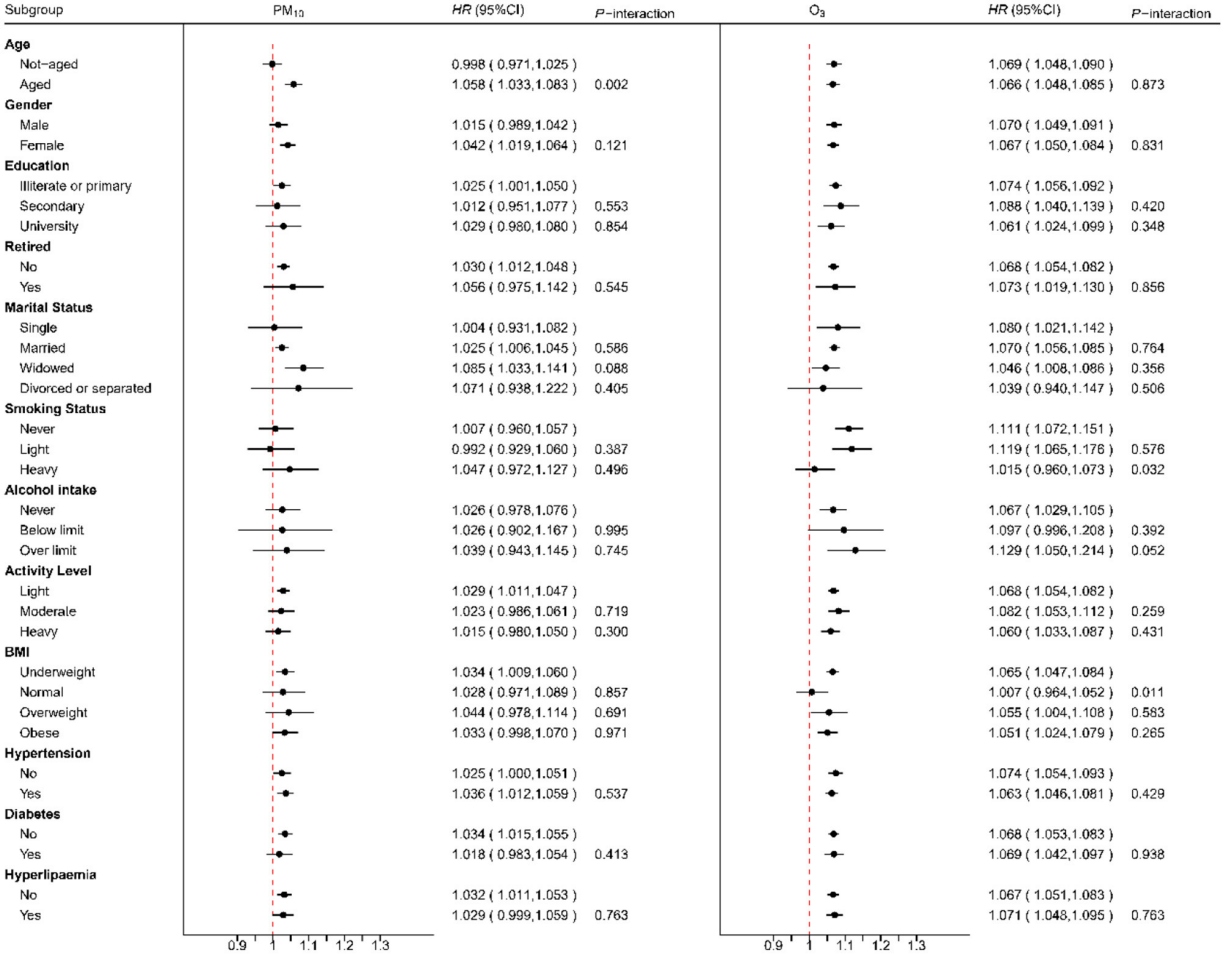
The modification effect on the association between air pollution (O_3_ and PM_10_) and all-cause hospitalization by different individual factors.

Figure 1 shows the moderating effect of covariates on the association between air pollution (i.e., O_3_ and PM_10_) and all-cause hospital admissions, respectively. For O_3_, we observed significant moderating effect of alcohol intake, BMI, and tobacco abuse. More specifically, participants who drank over limit (*HR* = 1.103, 95% *CI*: 1.006–1.211) were more susceptible to the impact of O_3_ than those who never drank (*HR* = 1.070, 95% *CI*: 1.056–1.084) or drank under limit (*HR* = 1.016, 95% *CI*: 0.960–1.076) with *P*_interaction_ being 0.052. In contrast, participants with normal BMI (*HR* = 1.072, 95% *CI*: 1.054–1.091) tended to be less susceptible than those who were underweight (*HR* = 1.105, 95% *CI*: 1.047–1.167), overweight (*HR* = 1.069, 95% *CI*: 1.045–1.094) or obese (*HR* = 1.017, 95% *CI*: 0.976–1.059) with *P*_interaction_ being 0.011. Similarly, participants who smoked heavily (*HR* = 1.071, 95% *CI*: 1.033–1.111) tended to be less susceptible than others (*P*_interaction_ = 0.032). As for PM_10_, we observed a significant effect of pollution among the older individuals (*HR* = 1.058, 95% *CI*: 1.033–1.083) but not among the others (*HR* = 0.998, 95% *CI*: 0.971–1.025) with *P*_interaction_ being 0.002.

Supplementary Figures S1–S4 show the moderating effect of individual factors on the associations between air pollution and cause-specific hospital admissions. For O_3_, non-aged people suffered from a higher risk of hospitalizations due to endocrine, nutritional and metabolic diseases (*HR*, 95%*CI*: non-aged, 1.158, 1.069–1.254 vs. aged, 1.069, 0.991–1.123; *P*_interaction_ = 0.154), digestive system diseases (*HR*, 95%*CI*: non-aged, 1.125, 1.060–1.194 vs. aged, 1.074, 1.014–1.137; *P*_interaction_ = 0.271) and circulatory system diseases (*HR*, 95%*CI*: non-aged, 1.093, 1.038–1.150 vs. aged, 1.020, 0.984–1.058; *P*_interaction_ = 0.060). In addition, underweight people were also more sensitive to the hospitalization due to these causes, *HRs* of underweight group and normal group for endocrine, nutritional & metabolic diseases were 1.114 (95% *CI*: 1.012–1.227) vs. 0.945 (95% *CI*: 0.752–1.186), for digestive system diseases were 1.086 (95% *CI*: 1.022–1.154) vs. 0.874 (95% *CI*: 0.748–1.020), for circulatory system diseases were 1.053 (95% *CI*: 1.014–1.094) vs. 1.012 (95% *CI*: 0.926–1.105), with *P*_interaction_ being 0.184, 0.004, and 0.412. For PM_10_, age and smoking status could modify its effect on the hospitalization due to some specific causes. Specifically, aged people had a higher risk to be admitted into hospital due to digestive system diseases with *HR* being 1.149 (95% *CI*: 1.063–1.242) compared with non-aged people with *P*_interaction_ being 0.004; people who smoked heavily had a significant higher risk for the hospital admission due to circulatory system diseases with *HR* being 1.291 (95% *CI*: 1.080–1.544) compared with lightly smoking people and non-smokers with *P*_interaction_ being 0.013.

## 4. Discussion

### 4.1. The association between air pollution and hospitalization

We found that the monthly concentration of PM_10_ and O_3_ exposures were positively associated with the risk of all-cause hospitalization. According to our estimates, increased O_3_ exposure was associated with an increased hospitalization due to each one of the multiple diseases except for respiratory system diseases. In contrast, increased PM_10_ exposure was only positively associated with the hospital admissions due to respiratory and digestive system diseases. Although few previous studies focused on the health effect of monthly exposure of air pollution, our results were in line with most of the evidence on the acute health effect. For example, a study conducted in southwestern France indicated that every 5 μg/m^3^ increment in the current-day exposure to O_3_ associated with a 5% increase in the risk of acute myocardial infarction hospitalization (22). Another study from Southern Europe suggested that each 14.4 μg/m^3^ elevation in PM_10_ at lag 0–5 days was associated with a 1.36% increase in the risk of respiratory hospitalization (23).

Air pollution may cause oxidative stress, inflammation, DNA damage and epigenetic changes (24), thereby triggering acute diseases or contributing to the increased rate of exacerbation of preexisting conditions, and leading to a greater risk of inpatient treatment. For instance, Li et al. found that each 10 μg/m^3^ increase in the 28-day moving average of O_3_ was correlated with a rise of 3.9% in total cholesterol, 8.2% in low-density lipoprotein cholesterol, 4.8% in Castelli risk indexes I and 7.0% in Castelli risk indexes II (25), and these markers are important risk factors of cardiovascular diseases (26). In addition, it has been widely reported that exposure to O_3_ elevated the level of systemic oxidative stress (27), which is one of the major contributors to the development of stomach diseases (28). The increase in short-term exposure to O_3_ was also observed to associate with the elevation of blood glucose and insulin level, and the occurrence of insulin resistance, which plays a vital role in the onset of type 2 diabetes (29). The mechanisms underlying the health effect of PM_10_ may be similar to that of O_3_ (30, 31).

### 4.2. The effect modification of individual factors on the association

We found people who drank over limit were more susceptible to the effect of air pollution on hospitalization. Although few evidence showed over drinking could strengthen the effect of air pollution for hospitalization, Dai et al. indicated that each 7% increase in the prevalence of heavy drinkers at county-level was associated with an increment of 40% in the estimated mortality effect of fine particles (32). The defense function of immune system is the main response to air pollution-induced inflammation for organism (33). However, a high alcohol intake is an essential risk factor for health which adversely affect the functioning of immune system (34, 35).

Surprisingly, we found people who smoked heavily were less vulnerable compared with those who smoked lightly and non-smokers. However, the result was supported by some existing findings. For example, Puett et al. suggested that current smokers had a lower risk of lung cancer associated with particulate matters compared with those who had never smoked or stopped smoking for more than 10 years (36). Tobacco smoke contains several chemical compounds and particulate matters, and was considered one of the major sources of indoor air pollution (37). Therefore, these heavy smokers might get used to air pollution already due to the years of tobacco-stimulation. Another possible reason is the “healthy smoker effect” which is similar to the well-known “healthy worker effect” (38), where the heavy smokers may be healthier than others because the unhealthy ones were more likely to have died or suffered from severe diseases, making them less likely to be included in this study.

We also found that people with abnormal body weight (i.e., underweight, overweight and obese) were more sensitive to the hospital admission associated with air pollution. For underweight people, evidence on their prolonged recovery duration after diseases were well documented (39), and underlying malnutrition in underweight people was a significant threat to their functional recovery (40). So, we speculated that the insufficient recovery capability from air pollution-induced damage may be a possible explanation for the greater susceptibility of underweight people to the effects of air pollution. For overweight and obese people, they had already been in the state of systemic oxidative stress. Air pollution-induced oxidative stress may aggravate their abnormal lipids metabolism (41) and thus attributes to the progression of diseases.

### 4.3. Limitations

We acknowledge that this study has some limitations, including the potential exposure misclassification by using the interpolation data of air pollution although the data was well validated against the observational data from monitoring stations. Another limitation is that the proportion of the elderly included in our study tended to be higher than the actual proportion reported for the area probably due to the fact the elderly were more likely to register at the Community Healthcare Centers and attended surveys on workdays. Our conclusions need to be extrapolated with cautions and validated with future studies based on more representative samples. Additionally, this is a single-city study which may limit the generalizing our findings. However, Guangzhou is the largest city in South China with a population of ~19 million from all over the country. It's of great significance to investigate the effect of long-term air pollution and identify corresponding modifiers in this area.

## 5. Conclusion

We found positive association of all-cause and cause-specific hospitalizations with monthly exposure to PM_10_ and O_3_. We also observed vulnerable populations to these pollution, such as people who drank over limit and those with abnormal body weight. Our results provided comprehensive evidence for the health effect of monthly exposure to air pollution. Clean-air Policies might be an effective way to reduced hospitalizations and specific measures should be taken to protect the most susceptible groups.

## Data availability statement

The raw data supporting the conclusions of this article will be made available by the authors, without undue reservation.

## Author contributions

WY, XJ, ZD, WZ, and YH contributed to conception and design of the study. WY, XJ, ZD, XW, and HC organized the database. WY, XJ, SC, YZ, GW, and WW performed the statistical analysis. WY, XJ, ZD, and WZ wrote the first draft of the manuscript. XL, YW, JJ, and WH wrote sections of the manuscript. All authors contributed to manuscript revision, read, and approved the submitted version.
