# Supplementary material for: The impact of monthly air pollution exposure and its interaction with individual factors: Insight from a large cohort study of comprehensive hospitalizations in Guangzhou area
Source: Front Public Health. 2023 Mar 21;11:1137196. doi: 10.3389/fpubh.2023.1137196 (PMC10071997; doi:10.3389/fpubh.2023.1137196)
Supplement: Supplementary file 1 [file Data_Sheet_1.pdf]

Appendix

Table S1 Cross validation results of Kriging interpolation for 4 main air pollutants in Guangdong Province.

Air pollutants	MAPE (%)	RMSE	*R* square
$PM_{2.5}$	10.04 (9.82,10.27)	3.97	0.90
PM_{10}	9.69 (9.50,9.88)	6.30	0.86
NO_2	18.55 (18.14,18.97)	6.17	0.81
O_3	9.34 (9.02,9.67)	10.54	0.82

Table S2 Correlation coefficient matrix of four main air pollutants exposure in a natural population cohort of Guangdong Province.

	$PM_{2.5}$	PM_{10}	NO_2	O_3
$PM_{2.5}$	1	——	——	——
PM_{10}	0.98	1	——	——
NO_2	0.88	0.91	1	——
O_3	0.03	0.02	-0.09	1

Table S3 The associations of air pollution, basic demographics, lifestyle information, and preexisting health conditions with all-cause hospitalization in single variable models.

**Variable**	**Categories**	***β***	***HR* (95%CI)**	***P***
**Air pollutant**				
O_3	——	0.078	1.081 (1.067,1.095)	<0.001
PM_{10}	——	0.023	1.023 (1.005,1.041)	0.001
$PM_{2.5}$	——	0.001	1.001 (0.967,1.036)	0.956
NO_2	——	0.024	1.024 (0.997,1.051)	0.077
**Basic demographics**				
Age	≤ 65	Ref		
	> 65	0.659	1.933 (1.822,2.051)	<0.001

Gender	Male	Ref		
	Female	-0.019	0.982 (0.929,1.037)	0.510
Ethnicity	Han	Ref		
	Others	-0.259	0.772 (0.532,1.119)	0.170
Education	Illiterate or primary	Ref		
	Secondary	-0.132	0.876 (0.815,0.943)	<0.001
	University or higher	-0.001	0.999 (0.943,1.058)	0.960
Retired	Yes	Ref		
	No	0.160	1.173 (1.031,1.335)	0.020
Marital Status	Single	Ref		
	Married	0.965	2.630 (2.320,2.971)	<0.001
	Widowed	1.129	3.093 (2.666,3.588)	<0.001
	Divorced or separated	1.068	2.910 (2.261,3.745)	<0.001
**Lifestyle Information**				
Smoking Status	Never	Ref		
	Light	0.017	1.017 (0.959,1.079)	0.565
	Heavy	0.107	1.113 (0.929,1.334)	0.245
Activity level	Light	Ref		
	Middle	-0.132	0.876 (0.832,0.922)	<0.001
	Heavy	-0.016	0.984 (0.941,1.029)	0.482
Alcohol intake	Never	Ref		
	Under limit	0.050	1.051 (0.907,1.218)	0.506
	Over limit	0.291	1.338 (1.175,1.523)	<0.001
**Preexisting health condition**				
BMI	Underweight	Ref		
	Normal	0.071	1.073 (1.019,1.131)	<0.001
	Overweight	0.299	1.348 (1.221,1.488)	<0.001
	Obese	-0.135	0.873 (0.807,0.945)	<0.001
Hypertension	No	Ref		

	Yes	0.490	1.632 (1.546,1.723)	<0.001
Diabetes	No	Ref		
	Yes	0.409	1.506 (1.410,1.608)	<0.001
Hyperlipidemia	No	Ref		
	Yes	0.206	1.229 (1.160,1.303)	<0.001

Table S4 Association between air pollution ($PM_{2.5}$ and O_3) and all-cause hospitalization risk.

	Single O_3 model	Single $PM_{2.5}$ model	Two-pollutant model	
			O_3	$PM_{2.5}$
	HR (95% *CI*)	*HR* (95% *CI*)	*HR* (95% *CI*)	*HR* (95% *CI*)
Model 1	1.081 (1.067,1.095)	1.001 (0.967,1.036)	1.081 (1.067,1.095)	0.997 (0.963,1.032)
Model 2	1.071 (1.058,1.085)	1.023 (0.989,1.058)	1.071 (1.057,1.084)	1.015 (0.981,1.049)
Model 3	1.071 (1.057,1.085)	1.023 (0.990,1.058)	1.071 (1.057,1.084)	1.015 (0.982,1.050)
Model 4	1.069 (1.056,1.083)	1.025 (0.991,1.059)	1.069 (1.055,1.082)	1.016 (0.983,1.051)

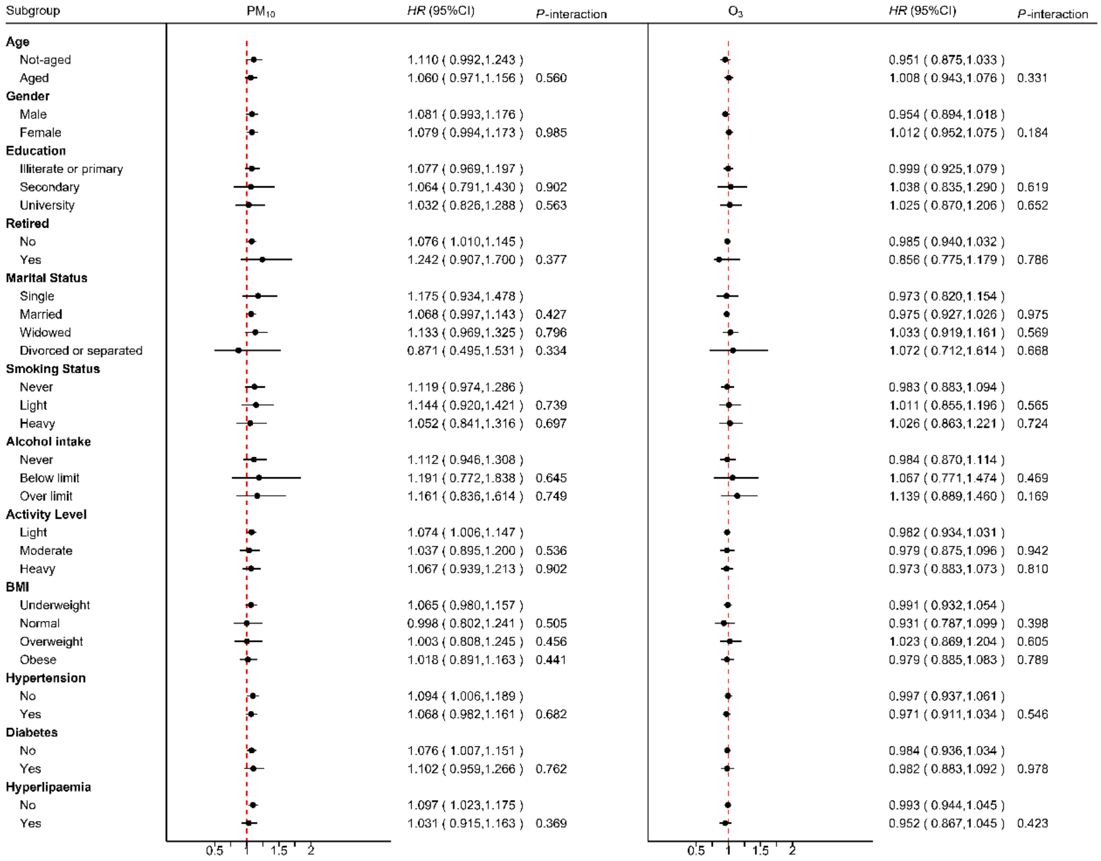

Figure S1. The modification effect on the association between air pollution (O_3 and PM_{10}) and hospitalization due to respiratory system diseases by different individual factors in the two-pollutant model.

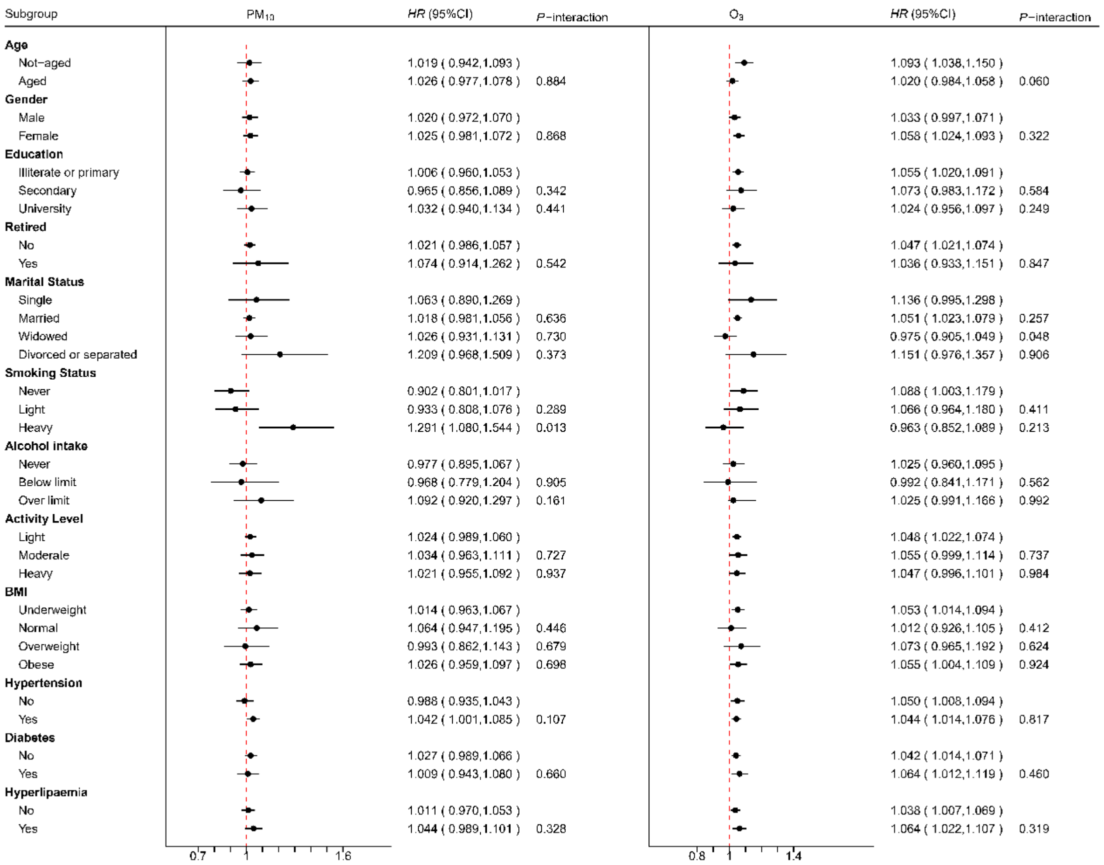

Figure S2. The modification effect on the association between air pollution (O_3 and PM_{10}) and hospitalization due to circulatory system diseases by different individual factors in the two-pollutant model.

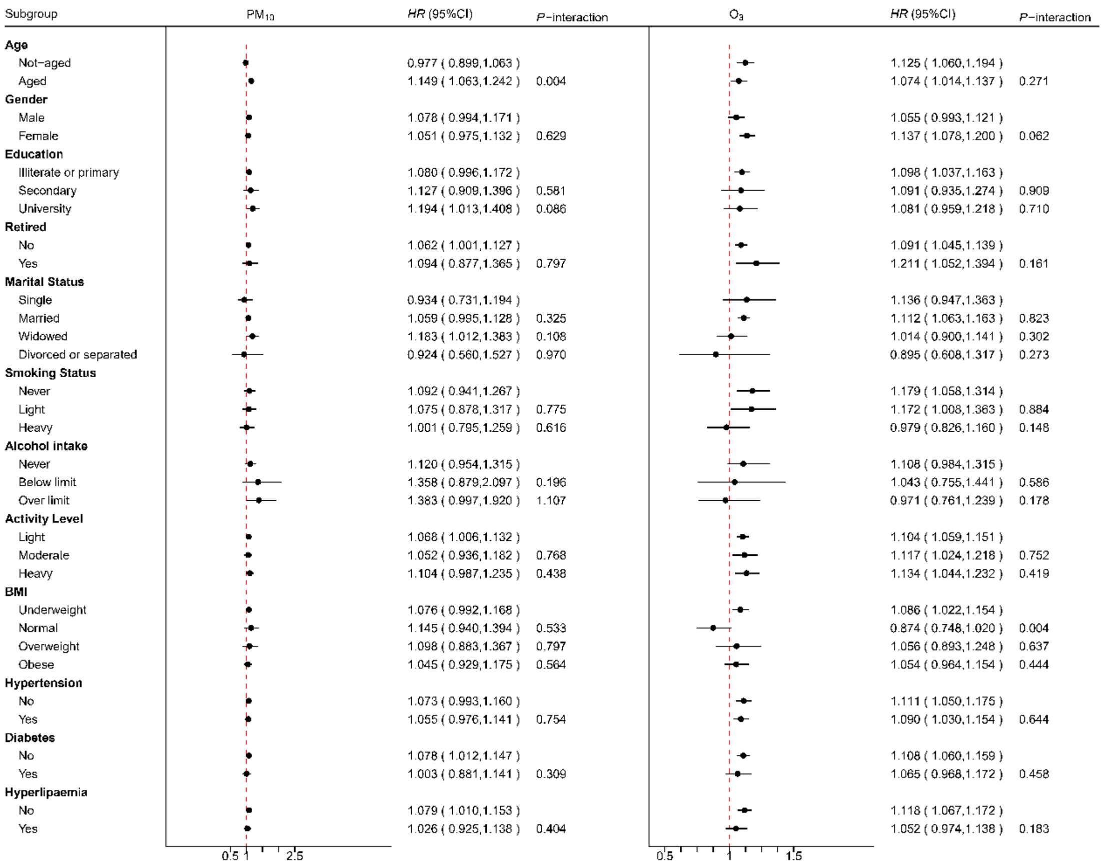

Figure S3. The modification effect on the association between air pollution (O_3 and PM_{10}) and hospitalization due to digestive system diseases by different individual factors in the two-pollutant model.

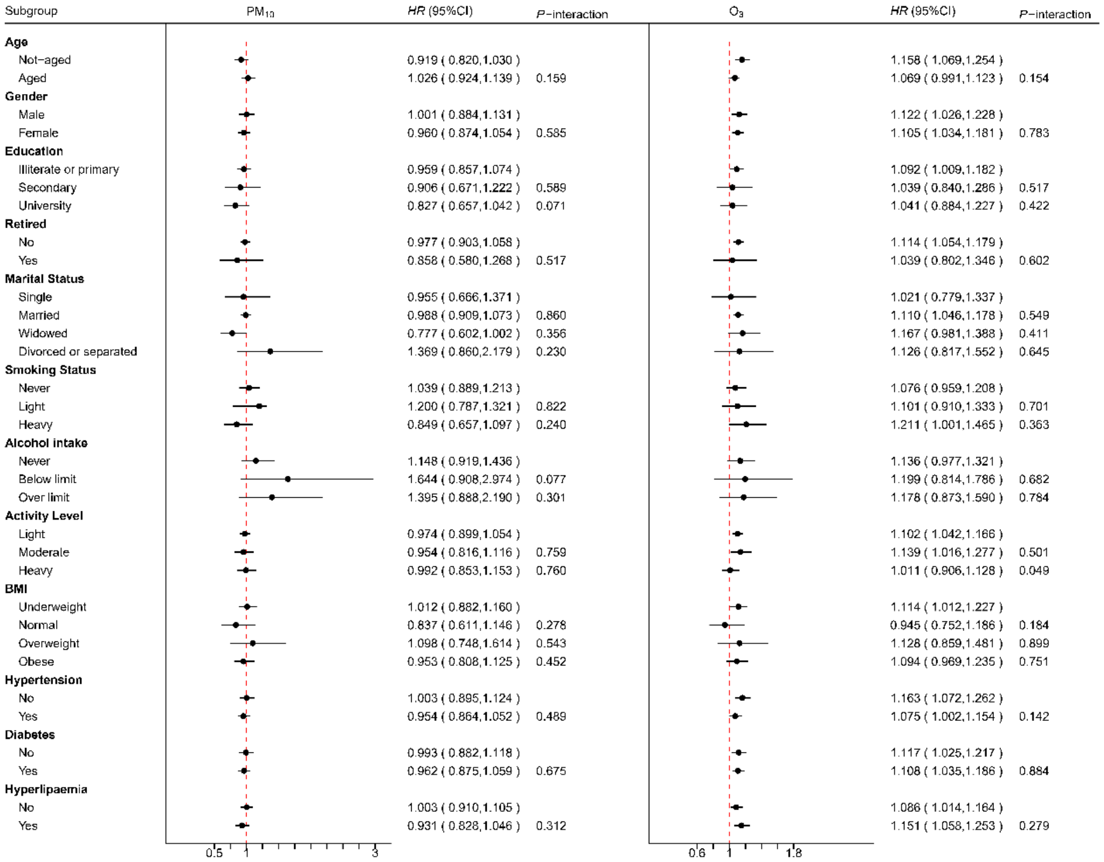

Figure S4. The modification effect on the association between air pollution (O_3 and PM_{10}) and hospitalization due to endocrine, nutritional & metabolic diseases by different individual factors in the two-pollutant model.

Table S5 Association between air pollution and all-cause hospitalization risk in sensitive analysis

	Single O_3 model	Single PM_{10} model	Two-pollutant model	
			O_3	PM_{10}
	HR (95% *CI*)	*HR* (95% *CI*)	*HR* (95% *CI*)	*HR* (95% *CI*)
Model 1	1.081 (1.067,1.095)	1.023 (1.005,1.041)	1.081 (1.067,1.094)	1.022 (1.004,1.040)
Model 2	1.071 (1.058,1.085)	1.033 (1.015,1.051)	1.070 (1.056,1.083)	1.030 (1.012,1.048)
Model 3	1.071 (1.058,1.085)	1.033 (1.015,1.051)	1.070 (1.056,1.083)	1.030 (1.012,1.048)
Model 4	1.069 (1.056,1.083)	1.034 (1.016,1.052)	1.068 (1.055,1.082)	1.031 (1.013,1.049)

Table S6 Association between air pollution and cause-specific hospitalization risk in sensitive analysis

Specific Cause	Single O_3 model	Single PM_{10} model	Two-pollutant model	
			O_3	PM_{10}
	HR (95% *CI*)	*HR* (95% *CI*)	*HR* (95% *CI*)	*HR* (95% *CI*)
Circulatory system diseases	1.048 (1.022,1.074)	1.025 (0.991,1.061)	1.047 (1.021,1.073)	1.023 (0.988,1.058)
Respiratory system diseases	0.985 (0.940,1.031)	1.080 (1.015,1.149)	0.984 (0.940,1.029)	1.080 (1.016,1.149)
Digestive system diseases	1.104 (1.059,1.151)	1.070 (1.011,1.133)	1.101 (1.057,1.147)	1.065 (1.005,1.128)
Endocrine, nutritional and metabolic diseases	1.109 (1.050,1.171)	0.981 (0.909,1.059)	1.111 (1.052,1.174)	0.973 (0.900,1.052)

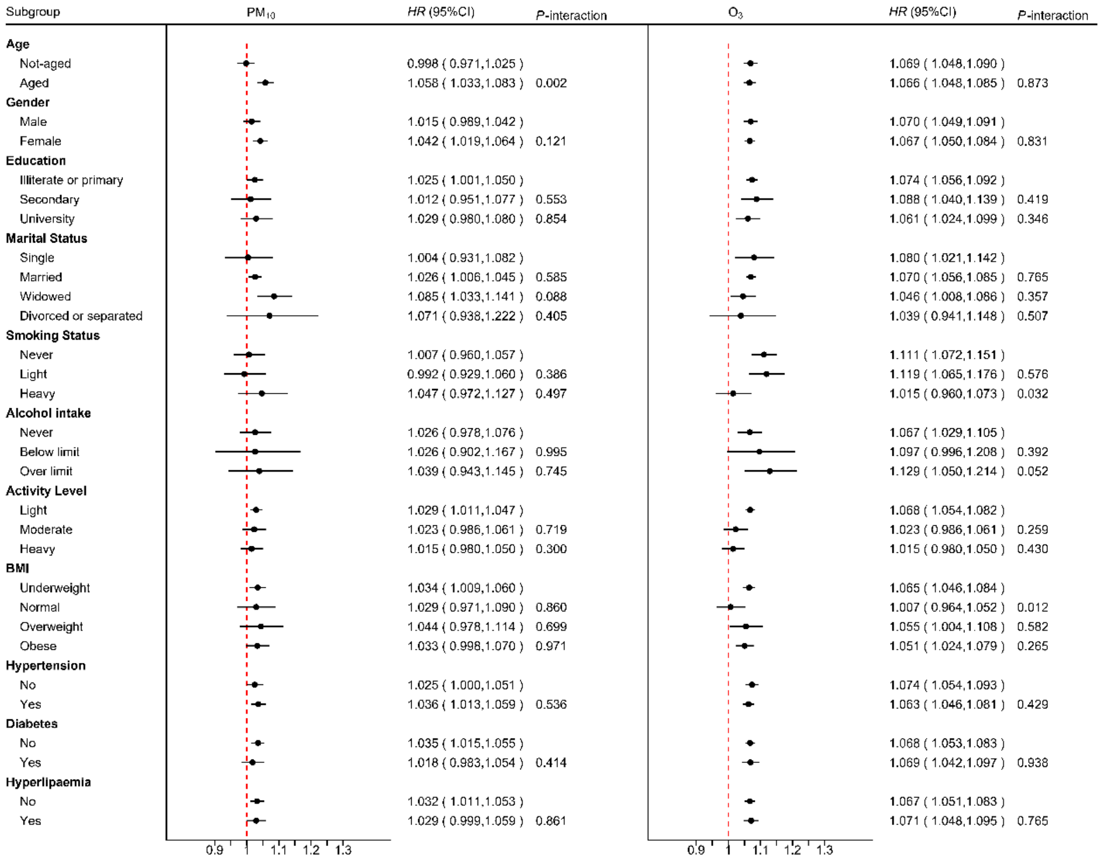

Figure S5 The modification effect on the association between air pollution (O_3 and PM_{10}) and all-cause hospitalization by different individual factors in sensitive analysis.

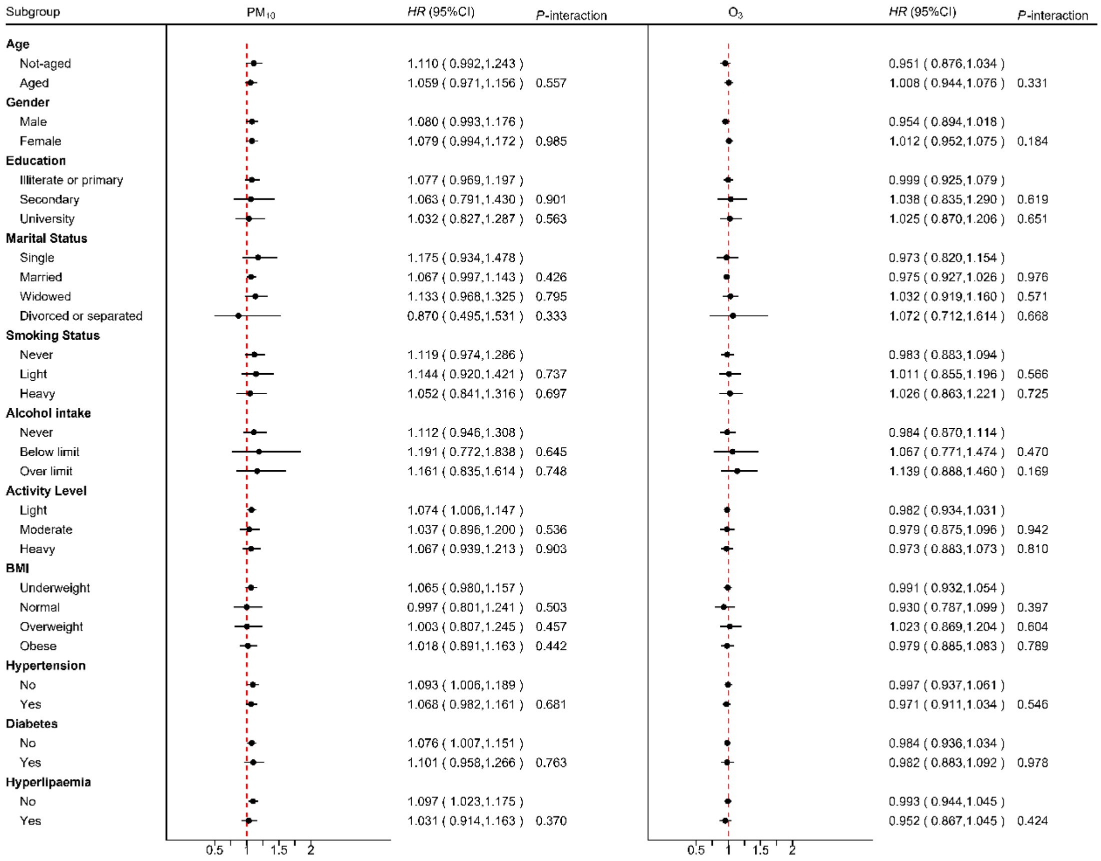

Figure S6. The modification effect on the association between air pollution (O_3 and PM_{10}) and hospitalization due to respiratory system diseases by different individual factors in sensitive analysis.

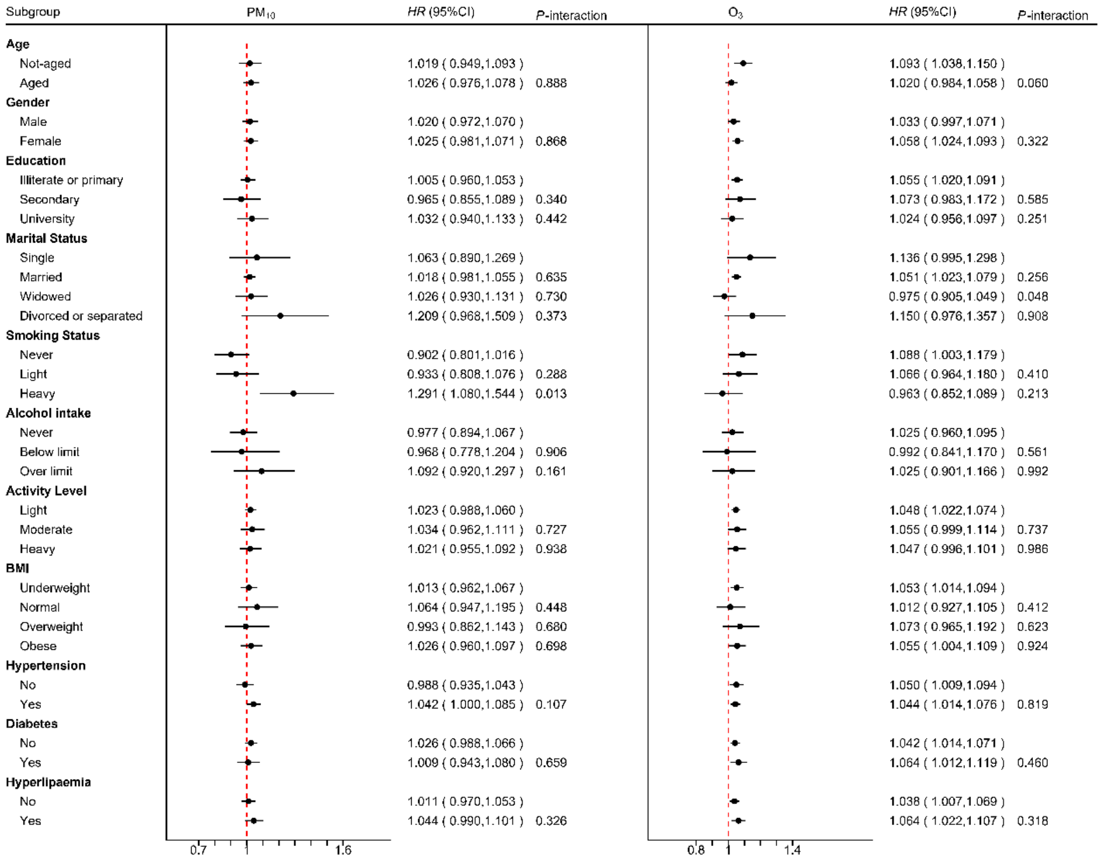

Figure S7. The modification effect on the association between air pollution (O_3 and PM_{10}) and hospitalization due to circulatory system diseases by different individual factors in sensitive analysis.

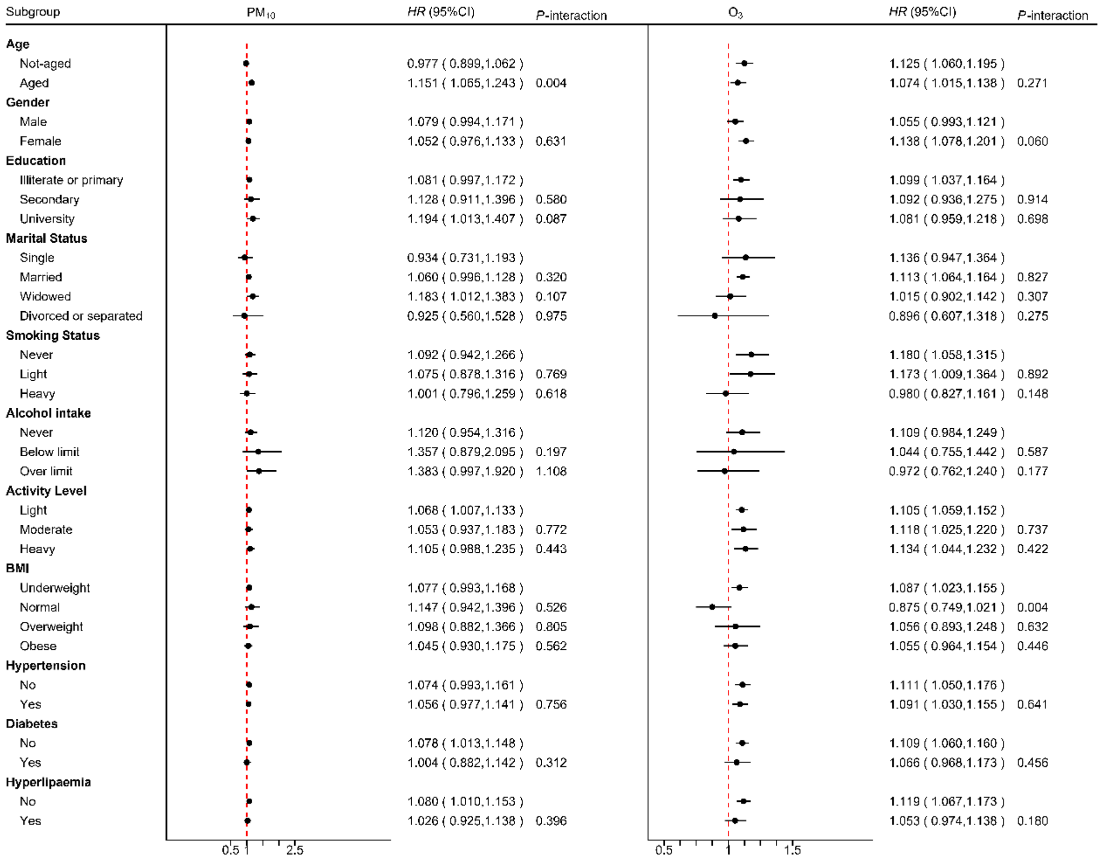

Figure S8. The modification effect on the association between air pollution (O_3 and PM_{10}) and hospitalization due to digestive system diseases by different individual factors in sensitive analysis.

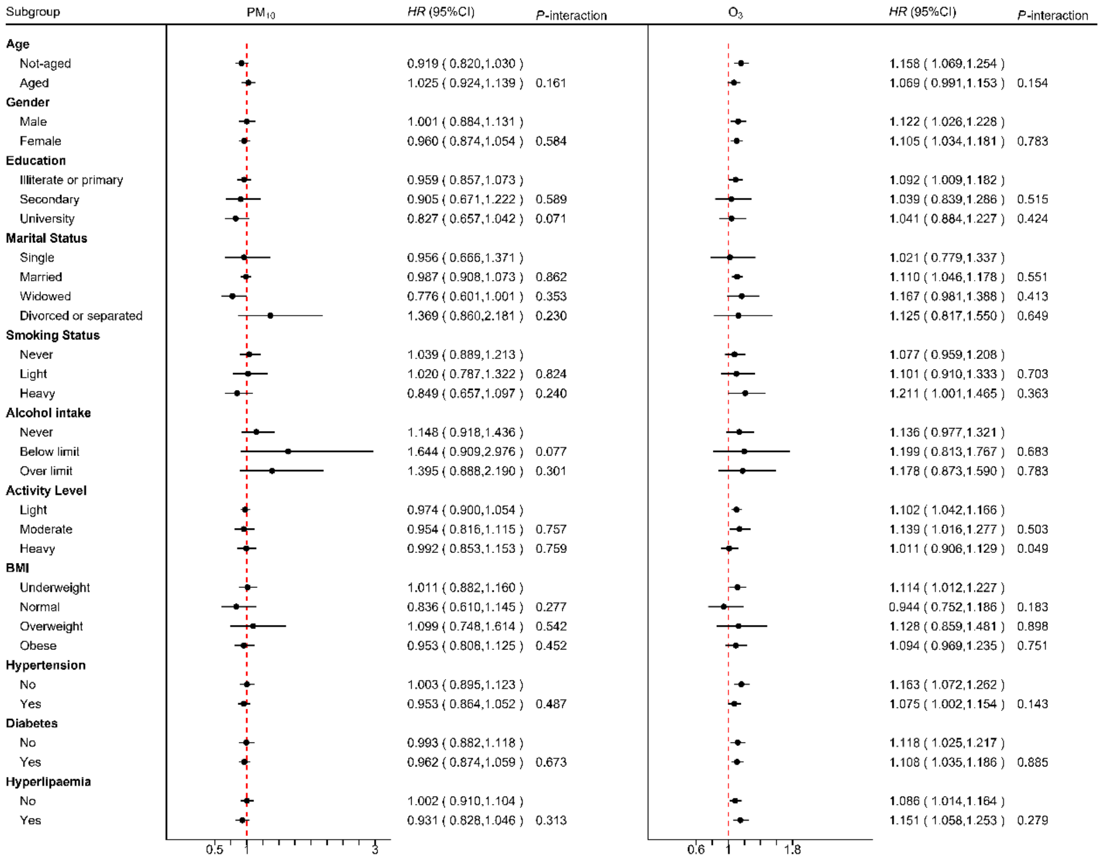

Figure S9. The modification effect on the association between air pollution (O_3 and PM_{10}) and hospitalization due to endocrine, nutritional & metabolic diseases by different individual factors in sensitive analysis.
